# The impact of neuropathic pain and other comorbidities on the quality of life in patients with diabetes

**DOI:** 10.1186/s12955-014-0171-7

**Published:** 2014-12-03

**Authors:** Vesna Dermanovic Dobrota, Pero Hrabac, Dinko Skegro, Ranko Smiljanic, Savko Dobrota, Ingrid Prkacin, Neva Brkljacic, Kristijan Peros, Martina Tomic, Vesna Lukinovic-Skudar, Vanja Basic Kes

**Affiliations:** Department of Diabetic Complication, Clinical Hospital Merkur, University Clinic Vuk Vrhovac, Dugi dol 4a, 10000 Zagreb, Croatia; Croatian Institute for Brain Research, School of Medicine, University of Zagreb, Salata 12, Zagreb, Croatia; Department of Internal Medicine, Clinical Hospital Merkur, Zajčeva 19, 10000 Zagreb, Croatia; Department of Diagnostic and Interventional Radiology, University hospital Center Zagreb, Kišpatićeva 12, 10000 Zagreb, Croatia; Department of Physiology, School of Medicine, University of Zagreb, Salata 3, 10000 Zagreb, Croatia; University Department of Neurology, University hospital Center “ Sestre milosrdnice“, Vinogradska 29, 10000 Zagreb, Croatia

**Keywords:** Diabetes, Diabetic polyneuropathy, Quality of life, Comorbidities

## Abstract

**Background:**

Diabetic polyneuropathy (DPN) is one of the most common complications of diabetes and can exist with or without neuropathic pain. We were interested in how neuropathic pain impairs the quality of life in diabetic patients and what is the role of comorbidities in this condition.

**Methods:**

The study included 80 patients with painful DPN (group “P”) and 80 patients with DPN, but without neuropathic pain (group “D”). Visual analogue scale (VAS) and Leeds assessment of neuropathic symptoms and signs (LANSS) pain scale were used for assessment of neuropathic pain, SF-36 standardized questionnaire for assessment of the quality of life and BDI questionnaire for assessment of depression.

**Results:**

Subjects in group P had statistically significantly lower values compared to group D in all 8 dimensions and both summary values of the SF-36 scale. We ascribe the extremely low results of all parameters of SF-36 scale in group P to painful diabetic polyneuropathy with its complications. The patients in group D showed higher average values in all dimension compared to group P, but also somewhat higher quality of life compared to general population of Croatia in 4 of 8 dimensions, namely vitality (VT), social functioning (SF), role-emotional (RE) and mental health (MH), which was unexpected result.

Clinically, the most pronounced differences between two groups were noted in sleeping disorders and problems regarding micturition and defecation , which were significantly more expressed in group P. The similar situation was with walking distance and color-doppler sonography of carotid arteries, which were significantly worse in group P. Consequently, subjects in group P were more medicated than the patients in group D, particularly with tramadol, antiepileptics and antidepressants.

**Conclusion:**

Painful DPN is a major factor that influences various aspects of quality of life in diabetic patients. Additionally, this study gives an overview of diabetic population in the Republic of Croatia, information that could prove useful in future studies.

## Background

Diabetes is one of the most common chronic non-infectious diseases and one of the leading public health problems of the modern society. It has high prevalence and ascending trend in the number of patients in all countries. Its late complications are the leading cause of increased morbidity and mortality of the patients and the main reason of increase of the cost of diabetes treatment [1].

According to the International Diabetes Federation (IDF), expected overall prevalence of diabetes in the year 2025 will be around 5.4%, a marked increase from 4% in 1995. Additionally, the largest number of diabetic patients, especially in developing countries, will come from the working population (people between 45 and 64 years of age), while in developed countries the majority patients will be above 65 years of age [2]. In Croatia (which is classified as developed country by King [2]), according to Croatian National Institute of Public Health (HZJZ) and the National Diabetes Registry CroDiab, the incidence of diabetes was 6.3% in the year 2011, with the total of 230.084 patients older than 18 years [3,4].

This tendency is reflected in the fact that diabetes is the sixth leading cause of mortality in United States and the seventh leading cause of mortality in Canada. The average life span is up to 15 years shorter in diabetic patients.

Diabetes is among ten leading causes of mortality in US [5] and Canada, significantly shortening the life span of patients [6] and also one of the most important risk factors in the development of cardiac and cerebrovascular diseases. Additionally, complications and comorbidities such as myocardial infarction, stroke, foot ulcer, amputation and blindness plague up to 50% of diabetic patients [7]. Complications significantly decrease the quality of life, reduce estimated life span and additionally increase the already increasing price of treatment of a diabetic patient [8]. This is why prevention and control of complications in diabetic patients is one of the most important mechanisms in any public health system.

One of the most common diabetic complications is diabetic polyneuropathy [9,10] which can exist with or without neuropathic pain. Its incidence is growing with the duration of diabetes. Around 50% patients with type II diabetes suffers from this condition, compared to approximately 30% in type I diabetes. Painful diabetic polyneuropathy occurs in 11-21% of patients with diabetes, with pain being one of the most common causes for seeking medical assistance. Such neuropathic pain often causes difficulties falling asleep, sleep disturbances caused by pain, burning sensations and itching. The loss of sleep often leads to anxiety and depression, thus additionally deteriorating sleeping disorders, so many patients enter a vicious cycle of sleep deprivation. Sleep deprivation in return results in lack of energy, strongly influencing patient’s ability to function through decreased motility and dependence on others in everyday functioning [11].

Diabetic polyneuropathy, both in its painful and non-painful form, significantly influences the patients’ quality of life. In this context, the aims of this investigation were to determine to what extent neuropathic pain (as one of the manifestations of diabetic polyneuropathy) impairs the quality of life of diabetic patients and do comorbidities additionally worsen the quality of life in this population.

In order to gain data applicable to our daily practice, and we believe also to practice in other similar clinics in Croatia and EU, we decided to use a random sample of diabetic population treated in our Clinic. This approach also helps in documenting the structure of diabetic population in Croatia in terms of age, sex, socioeconomic status etc.

## Methods

This investigation included 160 patients treated in Clinical hospital Merkur, University Clinic for Diabetes, Endocrinology and Metabolic Diseases “Vuk Vrhovac“ in period from May to October 2012. Included were subjects of both sexes, above 18 years of age, with the diagnosis of diabetes mellitus type I or II. Subjects meeting inclusion criteria were included consecutively, as they arrived to the Clinic, and were divided in two cohorts, depending on the presence or absence of neuropathic pain. The first group of 80 subjects consisted of patients with painful diabetic polyneuropathy, while the other group consisted of patients with diabetic polyneuropathy, but without pain. Diabetic polyneuropathy was diagnosed according to European Federation of Neurological Societies (EFNS) guidelines for treatment of neuropathic pain [12]. Pain at the moment of exclusion as well as within the past month was assessed by Visual analogue scale (VAS) and Leeds Assessment of Neuropathic Symptoms and Signs (LANSS) pain scale. The subjects with score of 12 or more on LANSS scale were included in the painful group, while the subjects with the score lower than 12 were included in the group of diabetic patients with DPN but without the painful component [13]. The study was approved by the authorised Ethics committee of Clinical Hospital Merkur, with all the subjects signing informed consent for participation in the study prior to inclusion.

Detailed medical history, followed by somatic and neurological status was taken from all subjects. Neurological examination included assessment of the muscle strength of the extremities, proprioceptive reflexes, loss of pressure sensation (10 g Semmes Weinstein monofilament was used to examine pressure sensation at hallux and metatarsal heads I, III and V) and vibration (by 128 Hz tuning fork) on lower limbs, as well as response to non-painful stimuli (by cotton swab), to examine the light touch sensation and presence of allodynia/hyperalgesia. This also included sonographic diagnosis of the arteries of the legs, electomyoneurography (EMNG) of upper and lower extremities and color-doppler flow imaging (CDFI) of carotid arteries. While taking medical history, a special focus was put on the existence of various comorbidities important for this study (Table 1), as well as demographic information (Table 2).

**Table 1:** **Comorbidity**

**Parameter**	**Group P**	**Group D**	**P**
**Vision impairment**			0.159
Reads with glasses	62 (82.7%)	63 (92.7%)	
Reads with magnifier	8 (10.7%)	4 (5.9%)	
Blind	5 (6.7%)	1 (1.5%)	
**Cardiac infarction**	4 (5.0%)	9 (11.3%)	0.148
**Angina pectoris**	20 (25.0%)	13 (16.3%)	0.172
**Coronary stent**	3 (3.8%)	11 (13.8%)	0.025
**Coronary bypass**	3 (3.8%)	3 (3.8%)	-
**Cerebrovascular stroke**	9 (11.3%)	4 (5.0%)	0.148
**ACI stenosis**	20 (25.0%)	10 (12.5%)	0.043
**ACI operation**	4 (5.0%)	3 (3.8%)	0.699
**Diabetic nephropathy**	8 (10.0%)	16 (20.0%)	0.076
**Dialysis**	5 (6.3%)	10 (12.5%)	0.175
**Leg ulcer**	7 (9.0%)	2 (2.6%)	0.089
**Operation on legs**	4 (5.1%)	4 (5.1%)	0.985
**Amputation**	6 (7.7%)	2 (2.6%)	0.152
**Sleeping disorders**	65 (81.3%)	12 (15.0%)	<0.001
**Micturition and defecation disorders**	18 (22.5%)	4 (5.0%)	0.001

**Table 2:** **Demographic and anamnestic data**

**Parameter**	**Group P**	**Group D**	***P***
**Sex, ratio of women (%)**	49 (61.3%)	28 (35.0%)	<0.001
**Age, years (SD)**	63.7 (8.4)	61.0 (10.6)	0.077
**BMI, kg/m** ^**2**^ **(SD)**	29.9 (5.7)	28.1 (4.3)	0.028
**Married (%)**	52 (65.0%)	61 (76.3%)	0.118
**Employed, N (%)**			
Yes	5 (6.3%)	19 (23.8%)	0.007
No	5 (6.3%)	3 (3.8%)	
Retired	70 (87.5%)	58 (72.5%)	
**Education, N (%)**			0.238
Elementary school	12 (15.0%)	6 (7.5%)	
High school	48 (60.0%)	48 (60.0%)	
Faculty	20 (25.1%)	26 (32.6%)	
**Members of household, N (%)**			0.363
1	14 (17.5%)	7 (8.8%)	
2	36 (45.0%)	36 (45.0%)	
3	11 (13.8%)	12 (15.0%)	
4 and more	19 (23.8%)	25 (31.3%)	
**Ratio of smokers, N (%)**	15 (18.8%)	15 (18.8%)	-
**Alcohol consumption, N (%)**	6 (7.5%)	17 (27.3%)	0.013
**Daily physical activity, hours (%)**			0.092
<1	46 (57.5%)	38 (47.5%)	
1-2	30 (37.5%)	30 (37.5%)	
2 and more	4 (5.0%)	12 (15.0%)	
**Financial situation satisfaction, N (%)**			<0.001
Yes	9 (11.3%)	25 (31.3%)	
Medium	47 (58.8%)	51 (63.8%)	
No	24 (30.0%)	4 (5.0%))	

Several other standardized questionnaires were used to assess quality of life, pain and mental status of the subjects. Quality of life was assessed by SF-36 (Short Form Health Survey) questionnaire, measuring 8 dimensions (Table 3), which can be additionally summarized to two standardized summary scores – PCS (Physical Component Summary) and MCS (Mental Component Summary). All mentioned parameters were calculated using methodology described by Taft [14], and compared to previously known results for the general Croatian population [15]. Aside from the two mentioned scales (LANSS and SF-36), Beck Depression Inventory (BDI) was also used to assess the presence and level of depressive disorder in subjects.

**Table 3:** **Quality of life and depression indicators**

**Parameter, points (SD)**	**Group P**	**Group D**	***p***
SF-36, PF*	28.0 (21.2)	61.9 (26.6)	<0.001
**SF-36, RP**	13.4 (30.0)	55.9 (42.5)	<0.001
**SF-36, BP**	30.8 (18.0)	57.0 (18.0)	<0.001
**SF-36, GH**	28.5 (17.8)	46.8 (18.8)	<0.001
**SF-36, VT**	39.9 (17.9)	61.0 (18.1)	<0.001
**SF-36, SF**	53.3 (27.5)	79.8 (20.6)	<0.001
**SF-36, RE**	43.3 (46.7)	74.2 (40.0)	<0.001
**SF-36, MH**	52.9 (20.7)	71.9 (17.6)	<0.001
**SF-36, PCS**	35.9 (6.2)	45.7 (8.2)	<0.001
**SF-36, MCS**	47.8 (9.6)	55.2 (8.0)	<0.001
**BDI**	19.1 (10.7)	9.6 (6.6)	<0.001

*Dimensions and summary scores of the SF-36 scale: PF = physical functioning; RP = role-physical; BP = bodily pain; GH = general health; VT = vitality; SF = social functioning; RE = role-emotional; MH = mental health; PCS = physical component summary; MCS = mental component summary.

All data was collected by study staff (physicians and nurses) that was blinded to the allocation of subjects to study groups. Scales used in the study were validated Croatian versions of scales, used in everyday clinical practice throughout the country (LANSS and BDI). In case of the SF-36, which is not used as extensively as the previously mentioned scales, validated translations and established norms previously published by Juresa [15] and Maslic [16] were used.

The primary aim of this study was to determine the quality of life of/in diabetic patients with painful diabetic polyneuropathy using MCS and PCS parameters of SF-36 scale, compared to the group of diabetic patients with diabetic polyneuropathy, but without painful manifestations. The SF-36 scale was used deliberately, although we are aware that there are other quality of life scales which are specific for diabetic patients. The main advantage of SF-36 is the existence of standardized values for the general population, making the comparison possible, which would be impossible with the disease-specific scales. The additional aim was to determine how comorbidity influenced the quality of life of diabetic patients. Rather than using two patient populations comparable in all parameters, a cross-sectional analysis based on the random population sample was used to obtain an objective insight into demographic, clinical and personal parameters of the two populations.

The needed number of subjects was calculated using NCSS PASS [17] software package with the following premises: statistical power (β) = 0.8; statistical significance (α) = 0.05; expected difference between groups = 5 value points of MCS or PCS parameters; expected standard deviation = 10 points on MCS or PCS scale. Sample size needed was 64 subjects per group. In order to decrease the influence of the variables unknown at the moment of sample calculation, the sample size was increased to 80 subjects.

Statistical analysis was done in Statistica software package (StatSoft, Inc., version 10). After testing for normality of distribution by means of Kolmogorov-Smirnov test, the appropriate parametric and non-parametric tests were used. For continuous variables, differences between two groups were tested by means of either Student t-test or Mann–Whitney U-test, while for multiple groups testing was done by ANOVA or Kruskal-Wallis ANOVA. For categorical variables testing was done by chi-square test. The values of categorical variables are represented as number and proportion of subjects, while for the variables measured on interval scale means and standard deviations are shown. The level of statistical significance was set to 0.05, which was in case of multiple comparisons interpreted using Bonferroni correction.

## Results

One hundred and sixty subjects included in the research were divided in two groups – diabetic patients with painful polyneuropathy (group “P”) and diabetic patients with polyneuropathy, but without painful sensations (group “D”). Basic demographic and clinical history data can be seen in Table 2., showing that two groups were comparable considering age, marital status, education, number of household members, smoking, BMI (after applying Bonferroni correction for multiple analyses), and the average time spent daily in physical activity. Group P had significantly higher female ratio (61.3%, versus 35.0%), while the employment rate was considerably lower (6.3%, versus 23.8%).

Regarding indicators of the primary illness, as well as the lipid profile parameters (Table 4), the two groups were comparable with the only exception being the type of diabetes, with somewhat lower proportion of type II diabetes in group D.

**Table 4:** **Basic data about the illness and lipid profile**

**Parameter**	**Group P**	**Group D**	**P**
**Diabetes, type II, N (%)**	75 (93.8%)	65 (81.3%)	0.017
**Duration of the illness, years (SD)**	16.0 (9.4)	19.0 (9.3)	0.176
**Insulin, N (%)**	58 (72.5%)	58 (72.5%)	-
**Antihypertensives, N (%)**	72 (90.0%)	71 (88.8%)	0.797
**Statins, N (%)**	70 (87.5%)	73 (91.3%)	0.442
**HbA1c, % (SD)**	7.1 (1.0)	7.3 (1.1)	0.342
**Cholesterol, mmol/L (SD)**	4.9 (1.2)	4.8 (1.1)	0.416
**HDL, mmol/L (SD)**	1.4 (0.4)	1.4 (0.4)	0.917
**LDL, mmol/L (SD)**	2.7 (0.9)	2.6 (0.8)	0.324
**Triglycerides, mmol/L (SD)**	1.8 (1.3)	1.8 (1.2)	0.710

The type and number of comorbidities is shown in Table 1. A higher proportion of ACI stenoses was found in group P, while more subjects with coronary stent were found in group D. However, the biggest (and statistically significant) differences were found in sleeping disorders and micturition and defecation disorders, which were both significantly more expressed in group P. The similar situation was with the walking distance and the results of the CDFI of carotid arteries, which were significantly worse in group P (Table 5). Expectedly, the subjects in group P were consuming more medications than the subjects in group D, which particularly refers to tramadol and antidepressants (Table 6).

**Table 5:** **Test results**

**Parameter**	**Group P**	**Group D**	**p**
**Walking distance**			0.003
<100 m	16 (20.0%)	6 (7.5%)	
100-500 m	59 (73.8%)	74 (92.5%)	
>500 m	5 (6.3%)	0 (0.0%)	
**Polyneuropathy**			0.436
No	0 (0.0%)	0 (0.0%)	
I	11 (14.1%)	16 (20.1%)	
II/III	67 (85.9%)	64 (80.0%)	
**EMNG**			<0.001
DPN	26 (32.5%)	56 (70.9%)	
Radiculopathy with DPN	54 (67.5%)	23 (29.1%)	
**Color-doppler of arteries**			0.218
Stenosis or occlusion	26 (32.5%)	19 (23.8%)	
Normal	54 (67.5%)	61 (76.3%)	
**Carotid color-doppler**			0.079
Normal	1 (1.3%)	0 (0.0%)	
Stenosis <50%	58 (72.5%)	69 (86.3%)	
Stenosis >50% or occlusion	21 (26.3%)	11 (13.8%)

**Table 6:** **Medication**

**Parameter**	**Group P**	**Group D**	**p**
**NSAR**	31 (38.8%)	30 (37.5%)	0.871
**Tramadol**	64 (80.0%)	33 (41.3%)	<0.001
**Antiepileptics**	7 (8.8%)	1 (1.3%)	0.029
**Antidepressants**	50 (62.5%)	30 (37.5%)	0.002
**Antispasmodics**	5 (6.3%)	2 (2.5%)	0.246
**Medication**			<0.001
Not every day	10 (12.5%)	62 (77.5%)	
1-2 a day	23 (28.8%)	17 (21.3%)	
3 and more a day	47 (58.8%)	1 (1.3%)	

**Figure 1:**
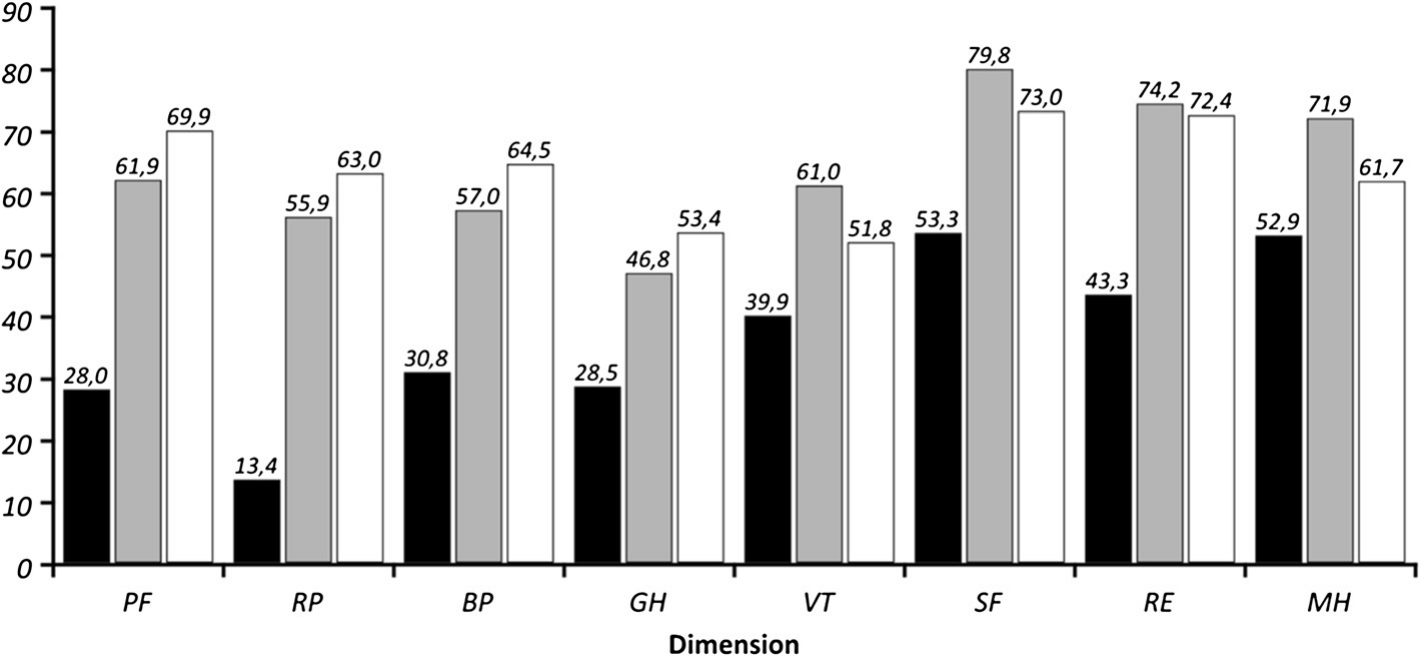
**Mean values of the dimensions of SF-36 scale for the group of diabetic patients with painful neuropathy (black), diabetic patients with painless neuropathy (grey) and random sample of population of Republic of Croatia (white) Physical functioning (PF); Role-physical (RP); Bodily pain (BP); General health (GH); Vitality (VT); Social functioning (SF); Mental Health (MH); Role-emotional (RE).**

Table 3 shows the parameters of the quality of life, expressed through 8 dimensions and two summary values of SF-36 scale. Figure 1 shows the same data, together with the results obtained from a random sample of 5.048 people of general population of the Republic of Croatia [15]. Besides the (expected) statistically significant differences between groups P and D in all 8 dimensions and both summary values of SF-36 scale, it is interesting to notice that subjects in group D had higher average values in 4 of 8 dimensions of the scale compared to the general population of the Republic of Croatia. Additionally, in the same table the mean values of BDI scale are shown, indicating significantly higher level of depression in group P.

Apart from the above mentioned differences, the differences in sex were examined in the whole sample (N = 160; 83 male, 77 female). It was shown that women, although comparable to men in terms of employment status (p = 0.131), were on average significantly less educated than men (p = 0.001), and were significantly less satisfied with their financial situation (p = 0.007). These differences were not attributable to age, since men and women were comparable in that criterion (p = 0.298), as well as to the type of diabetes (p = 0.256) or the presence of comorbidities (p = 0.158). Additionally, men and women were comparable in all the categories of comorbidities, except for bypass (6 men and none of the women; p = 0.016) and amputation (7 men, 1 woman; p = 0.037). It is also interesting that men had higher quality of life, based on both summary values of SF-36 scale, namely 43.3 versus 38.2 for PCS (p < 0.001) and 52.8 versus 50.1 for MCS (p = 0.072).

Another analysis performed on the whole sample (N = 160), was the analysis of impact of comorbidities on the quality of life (Table 7). Of 15 comorbidities already discussed earlier (see Table 1), five (coronary stent, coronary bypass, ACI stenosis, leg ulcer and amputation) had no impact on any of the dimensions and summary scores assessed by the SF-36 scale. Presence of any of the remaining 10 comorbidities has led to significantly lower values in different dimensions of the SF-36 scale. Two comorbidities (cerebrovascular stroke and operation on legs) impacted only one dimension. Conditions affecting numerous categories were sleeping disorders, micturition and defecation disorders, angina pectoris and blindness. Dimensions of the SF-36 scale affected by most comorbidities were physical functioning, general health and physical component summary.

**Table 7:** **Effect of comorbidities on the quality of life in all study subjects (N = 160)**

**Parameter** ^**§**^	**BL**	**CI**	**AP**	**CT**	**CB**	**CS**	**AS**	**AO**	**DN**	**DI**	**LU**	**OL**	**AM**	**SD**	**MD**
**SF-36, PF**	*	*	**					*				*		**	**
**SF-36, RP**	*		**											**	**
**SF-36, BP**			**											**	**
**SF-36, GH**	*		**			*		**	*	*				**	**
**SF-36, VT**			**											**	**
**SF-36, SF**			*											**	**
**SF-36, RE**														**	**
**SF-36, MH**									*					**	**
**SF-36, PCS**	**	*	**					*	*	*	*			**	**
**SF-36, MCS**														**	**

*^§^Description of SF-36 dimensions and summary scores can be found in legend for Table 6.

^**§**^BL = blind; CI = cardiac infarction; AP = angina pectoris; CT = coronary stent; CB = coronary bypass; CS = cerebrovascular stroke; AS = ACI stenosis; AO = ACI operation; DN = diabetic nephropathy; DI = dialysis; LU = leg ulcer; OL = operation on legs; AM = amputation; SD = sleeping disorder; MD = micturition and defecation disorders.

Legend for table cells: empty = no statistical significance; * = p<0,01; ** = p<0,001.

## Discussion

As stated earlier, subjects were included in the study in the order of arriving to our clinic. Since there was no impact on the subject selection by the examiners, a certain misbalance between the groups regarding age, sex and other parameters can be noticed (Table 2). Difference in sex is especially important because from our experience as well as from the already published results [18], diabetic women tend to have lower level of education compared to men of the same age and same health status. We hypothesize that this may lead to lower income and/or worse general workplace conditions for women and consequently lower satisfaction with their financial status as well. It is also found that men generally have subjectively higher quality of life than women [19], which is again confirmed in our population. One of possible explanations is that the higher level of education, associated with greater awareness of the disease and lifestyle choices, results in more active and effective acceptance of the disease. Besides diabetes [20,21], such concept is documented in variety of cultures and conditions [22-25]. This initiates a positive feedback with more active approach to the treatment of diabetes, better glycaemic control, and less serious complications. In this context, our experience has also shown that during the obligatory five-day education in Day hospital, where patients receive basic knowledge about diabetes and its complications through a multidisciplinary approach of a support psychologist, psychiatrist and diabetologist, patients with better education accept the facts about the disease easier and faster compared to less educated patients.

Considering our field of interest, the walking distance was of a special concern, being one of the main indicators of physical health of diabetic patients [26,27]. The group with painful polyneuropathy expectedly had significantly higher proportion of subjects with short walking distance (up to 100 m). However, in the same group there were a number of subjects (namely 5 persons) with walking distance longer than 500 m, whilst in the group of diabetic patients without painful neuropathy there were no such subjects. This seemingly surprising result is explicable by previously mentioned good level of disease awareness, learned by our patients during the Day-hospital education. Therefore, although sensing pain, patients are taught to continue to walk to alleviate development of collateral circulation in legs. Special clinical challenge for a neurologist is the presence of radiculopathy in a high percentage of subjects in the painful group, which is also unexpected; i.e. it was considered that diabetic patients had reduced walking distance exclusively due to claudications [28,29] caused by stenotic changes of the lower extremity arteries. Our results shine a new light on the cause of this problem, which is particularly important because there have been no similar surveys in the diabetic population in Croatia so far.

Bearing in mind the primary goal of this research, i.e. the quality of life of the two groups of diabetic patients, a significant difference between subjects with painful diabetic polyneuropathy and subjects without painful neuropathy is clearly evident. Additionally, both groups show values significantly different from the general population. The quality of life is expectedly lower in the painful group than in the group of subjects with DPN without pain. We believe that there is a number of reasons for that. Manifestations of painful polyneuropathy, associated with chronic back pain, can exhaust the patient more than the primary illness (diabetes), and these conditions are of great importance for functioning of a diabetic patient and his/her mental status. These conditions require an additional effort in terms of time and energy needed for treatment, and lay a burden on subject’s financial resources in terms of medications and additional medical treatment. These elements, basically caused by painful symptoms, may adversely affect the overall quality of life.

The other important factor is mental health. It reflects on the quality of life of subjects with painful polyneuropathy as lower value of cumulative parameter MCS, as well as higher level of depression according to BDI scale. This is expected, both in terms of the higher incidence of depression in diabetics in general [30] and in higher intensity of depressive symptoms in group with painful neuropathy [31]. In our opinion, and in line with some previously published results [32,18], depression is a major contributor impairing quality of life in diabetics.

Sleeping disorders are drastically more common in the painful group. With its particular influence they additionally deteriorate the condition of these subjects. This vicious circle is intensified by the fact that painful diabetic polyneuropathy is very difficult to treat, and 30-40% of patients do not respond to any kind of therapy [33], thus increasing the overall cost of treatment [33]. In this context, some recent studies [34] show patients more often neglect the treatment of the primary disease, like diabetes or hypertension, and rather take medications for pain treatment, e.g. analgesics. This is confirmed in our study as well, with significantly higher proportion of subjects taking multiple medications in the painful group, especially analgesics (Table 6). Alternatively, it is possible that increased medication results in lower alcohol consumption in the same group (Table 2).

As we have shown with the analysis of the effects of comorbidities on the quality of life (Table 7), low values of all SF-36 scale parameters in group P can be (in part, at least) explained by higher prevalence of comorbidities in the P group. This is especially true for sleeping disorders and micturition/defecation disorders, both significantly lowering all dimensions of SF-36 scale and both significantly more prominent in P group. While negative effects of sleep disturbances on quality of life are well documented [35], the micturition/defecation disorders’ effects are described mostly in surgical patients. Their consistent and statistically highly significant effect on all dimensions of SF-36 scale is an interesting finding which we hope will be confirmed by other researchers.

Subjects in group D have higher mean values in all SF-36 parameters compared to group P, but also show somewhat higher quality of life compared to general population in Croatia in 4 of 8 dimensions, namely vitality (VT), social functioning (SF), role-emotional (RE) and mental health (MH), which was an unexpected result. Our hypothesis is that this outcome was a result of a significantly bigger portion of male subjects, as well as those with high education in group D versus group P, but also versus general population from year 2000 [15]. The proportion of the highly educated in Croatia in year 2001 was 12.0%, and in 2011 the proportion increased to 16.4%. In the year 2011 the proportion of the highly educated of the age 60–64, which is the age of our subjects, was 20% for men and 16.2% for women. The said proportions were in a distinct opposition with subjects in group P, where proportion of the highly educated was 25.1%, as well as in group D where the same proportion was 32.6% [3].

Finally, quality of life of diabetic patients is one of the most important aspects of the disease, both because of its effect on the long-term prognosis [36], and on the economic burden of the disease [7]. Neuropathic pain, besides its direct incapacitating effect on patient, shows additional negative effect by significantly lowering the quality of life. Treatment of neuropathic pain can therefore have multiple positive effects on such patients.
